# Afferent loop syndrome in a patient having incomplete annular pancreas

**DOI:** 10.1259/bjrcr.20160015

**Published:** 2016-09-03

**Authors:** Warun Jose, Deepali Saxena, Ravi Hoisala

**Affiliations:** Department of Radiodiagnosis, St. John's Medical College, Bangalore, India

## Abstract

Afferent loop syndrome is a rare complication following a Billroth II procedure and is seen in 3/1000 cases. This case report shows the importance of radiological imaging in a patient with a past history of abdominal surgery, for which no records were available. Imaging showed incomplete annular pancreas, leading to narrowing of the second part of the duodenum, which was the indication for gastrojejunostomy in the past, now presenting with features of afferent loop syndrome.

## Background

Afferent loop syndrome (ALS) is a rare complication of Billroth II gastrojejunostomy, Roux-en-Y gastroenterotomy and Whipple's operation. Most cases of ALS are caused by obstruction owing to adhesions, kinking at the anastomosis site, internal hernia, stomal stenosis, malignancy or inflammation surrounding the anastomosis. Clinically, ALS is often difficult to diagnose because its presentation may be vague and non-specific. Delayed diagnosis may result in life-threatening events such as bowel ischaemia or perforation.^1^ Therefore, prompt imaging using conventional barium studies and contrast-enhanced CT scan of the abdomen is needed to suggest the diagnosis of ALS and decide on further management.

## Clinical presentation

A 70-year-old male presented with multiple episodes of loose stools and vomiting for the past 3 months, which was managed conservatively. The patient gave a history of having undergone an abdominal surgery 20 years ago for clinical features suggestive of gastric outlet obstruction. No surgical records were available. No other significant history was noted.

On general examination, the patient was conscious, afebrile, normotensive, with a pulse rate of 76  min^–1^ and without signs of dehydration. A right paramedian abdominal scar and mild abdominal distension were noted. There was no tenderness or organomegaly. Bowel sounds were normal. Laboratory investigations revealed haemoglobin 10.2 g/dl, normal blood counts, serum Na+ 131 mEq l^−1^ and K+ 4 mEq l^−1^, serum creatinine 0.6 mg dl^−1^ and serum albumin 2.4 g dl^−1^. Liver parameters were within normal limits.

The initial differential diagnosis was broad and included abdominal abscess, peptic ulcer disease, gastric outlet obstruction, ALS, strictures, hernias, mesenteric ischaemia and bacterial overgrowth syndrome.

Upper gastrointestinal endoscopy showed normal appearance of the gastrojejunal stoma. The afferent loop of the bowel was dilated proximal to the stoma and distended with fluid residue.

**Figure 1.**
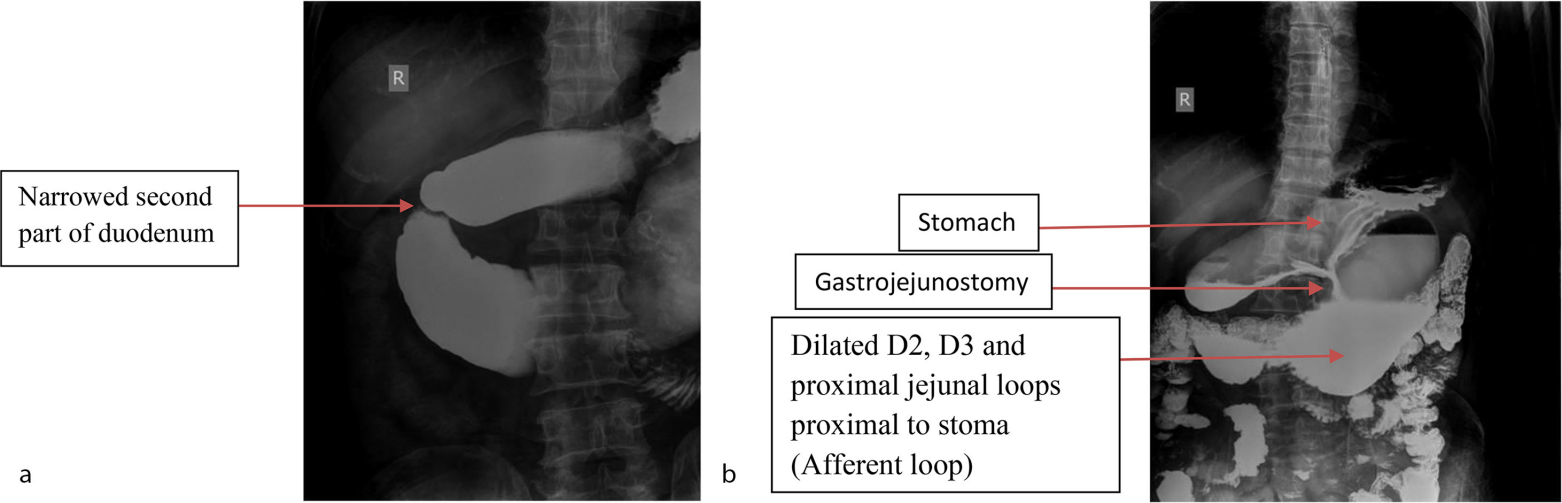
(a) Barium meal follow through image demonstrating narrowing of the second part of the duodenum. (b) Barium meal follow through image demonstrates gastrojejunostomy with dilatation of part of the D2, D3 and D4 segments of the duodenum and proximal jejunal loops, with passage of contrast distally.

On barium meal follow through, a gastrojejunostomy stoma was demonstrated. Additionally, abrupt narrowing of the D2 segment of the duodenum was noted with no mucosal irregularity. The contrast was seen to pass through the stoma and retrogradely fill the dilated D3, D4 and jejunal loops proximal to the stoma opening. The contrast was seen to pass into the rest of the jejunal loops (Figure 1a,b).

**Figure 2.**
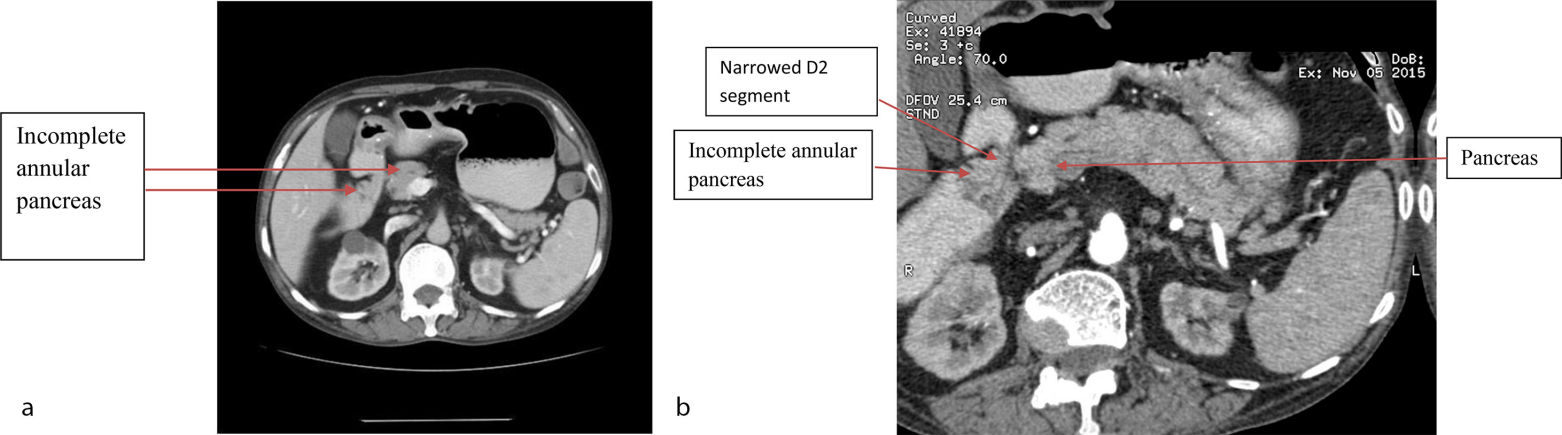
(a) Axial contrast-enhanced CT scan of the abdomen (with oral and intravenous contrast) demonstrating incomplete annular pancreas narrowing the D2 segment of the duodenum. (b) Axial contrast-enhanced CT scan of the abdomen with curved reformatting (with oral and intravenous contrast) demonstrating incomplete annular pancreas narrowing the D2 segment of the duodenum.

**Figure 3.**
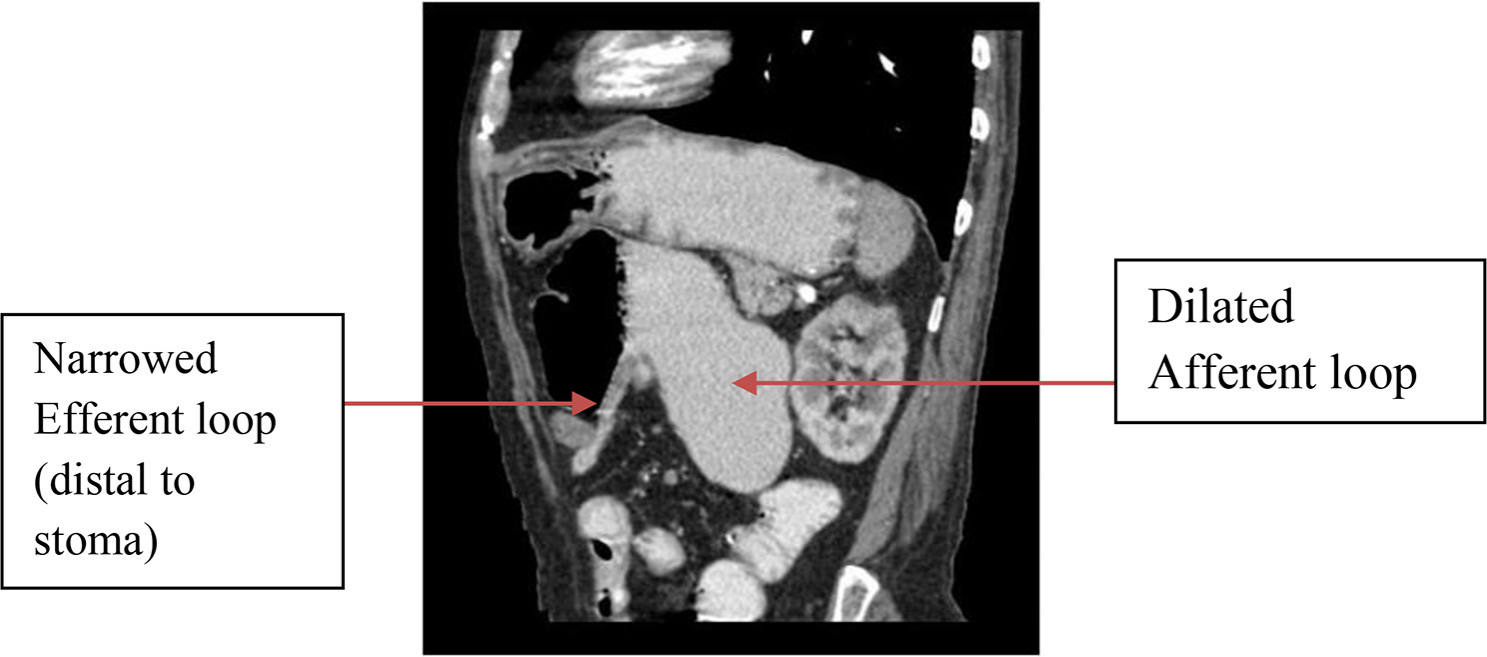
(a) Coronal contrast-enhanced CT image of the abdomen demonstrates narrowing of the loop of jejunum distal to the gastrojejunal stoma with dilatation of the afferent loop (D2, D3, D4 and jejunal loops proximal to the stoma.)

Contrast-enhanced CT (CECT) scan of the abdomen was performed for further evaluation of the narrowing in the D2 segment of the duodenum and the stoma site. On CECT of the abdomen, the cause of narrowing of the D2 segment was seen to be the incomplete annular pancreas. Narrowing of a short segment of the jejunum (distal to the gastrojejunal stoma) with dilatation of the intervening D2, D3 and D4 segments of the duodenum and the proximal jejunal loop was noted, suggesting afferent loop dilatation (Figures 2a,b and 3a).

## Treatment

The patient was initially managed with nasogastric tube drainage with temporary relief of symptoms, partial parenteral nutrition and probiotics, following which his general health improved. Surgical correction or luminal stent placement was offered to the patient, but the patient deferred the procedure. Subsequently, the patient was lost to follow-up.

## Discussion

Proximal or afferent loop consists of the oversewn duodenal stump and the most proximal jejunum, which carry the biliopancreatic secretions. The distal or efferent loop extends downstream, carrying gastric contents distally.^2^ ALS is caused by three different mechanisms, which include mechanical obstruction of the afferent loop, preferential gastric emptying into the afferent loop and obstruction of the efferent loop resulting in preferential filling of the afferent loop. This condition occurs infrequently following gastroenterotomy reconstruction.^1^ ALS can be acute or chronic. The clinical features include abdominal pain, nausea and vomiting, postprandial fullness and, rarely, post-obstructive jaundice.^3^ In the case presented, the clinical presentation had multiple differentials, including peptic ulcer disease, gastritis, gastric outlet obstruction, small bowel obstruction, mesenteric ischaemia and ALS. Radiographic barium examination findings of ALS include non-filling of the afferent limb or preferential filling and retention of barium in a dilated afferent limb for at least 60 min.^3^ Fluid-filled C-shaped afferent loop (C-loop appearance) on coronal plane in combination with valvulae conniventes projecting into the lumen (keyboard sign) is the most common multidetector CT feature of ALS.^1^ Complications of ALS such as a dilated gallbladder, biliary dilatation and pancreatitis also are identified readily on CT and ultrasound imaging.^3^

Annular pancreas occurs in 1/20,000 of the population and results from failure of the ventral bud to rotate with the duodenum, causing encasement of the duodenum. This encasement of the duodenum by a band of pancreatic tissue can be complete (pancreatic parenchyma or annular duct completely surrounds the duodenum) or incomplete (partial encasement of the duodenum). In a patient with gastric outlet obstruction, pancreatic tissue extending in a posterolateral or anterolateral direction to the second part of the duodenum or pancreatic tissue anterior and posterior to the duodenum should raise the suspicion of annular pancreas.^4^

In the case discussed above, the patient had narrowing of the efferent loop of the jejunum, leading to preferential filling of the afferent loop. Since there is narrowing of the D2 segment of the duodenum (incomplete annular pancreas) proximal to the afferent loop, it leads to progressive dilatation of the afferent loop, leading to ALS.

## Learning points

1. In a patient with past history of gastrojejunostomy presenting with abdominal pain, distension and vomiting, ALS should be considered in the differential diagnosis.
2. Prompt imaging with conventional barium meal follow through and CECT of the abdomen with oral contrast is required, as surgery is usually necessary to relieve the mechanical obstruction and possibly revise the gastrojejunostomy anastomosis.

## Consent

Informed consent to publish this case was obtained and is held on record. Patient's identity has not been disclosed in the case report.
